# Examining Tweet Content and Engagement of Users With Tweets About Hikikomori in Japanese: Mixed Methods Study of Social Withdrawal

**DOI:** 10.2196/31175

**Published:** 2022-01-11

**Authors:** Victor Pereira-Sanchez, Miguel Angel Alvarez-Mon, Toru Horinouchi, Ryo Kawagishi, Marcus P J Tan, Elizabeth R Hooker, Melchor Alvarez-Mon, Alan R Teo

**Affiliations:** 1 Department of Child and Adolescent Psychiatry NYU Grossman School of Medicine New York, NY United States; 2 Department of Psychiatry Columbia University New York, NY United States; 3 Division of Translational Epidemiology New York State Psychiatric Institute New York, NY United States; 4 Department of Psychiatry and Clinical Psychology Clinica Universidad de Navarra Pamplona Spain; 5 Department of Medicine and Medical Specialties Faculty of Medicine and Health Sciences University of Alcala Alcala de Henares Spain; 6 Department of Psychiatry and Mental Health Hospital Infanta Leonor Madrid Spain; 7 Department of Psychiatry and Neurology Hokkaido University Graduate School of Medicine Hokkaido Japan; 8 Department of Psychiatry Chiba Psychiatric Medical Center Chiba Japan; 9 Department of Child and Adolescent Psychiatry South London and Maudsley NHS Foundation Trust London United Kingdom; 10 VA Portland Health Care System Health Services Research & Development Center to Improve Veteran Involvement in Care Portland, OR United States; 11 Immune System Diseases-Rheumatology, Oncology Service and Internal Medicine Hospital Universitario Principe de Asturias Alcalá de Henares Spain; 12 Department of Psychiatry Oregon Health & Science University Portland, OR United States

**Keywords:** hikikomori, loneliness, social isolation, social withdrawal, Twitter, hidden youth, mobile phone

## Abstract

**Background:**

Hikikomori is a form of severe social withdrawal that is particularly prevalent in Japan. Social media posts offer insight into public perceptions of mental health conditions and may also inform strategies to identify, engage, and support hard-to-reach patient populations such as individuals affected by hikikomori.

**Objective:**

In this study, we seek to identify the types of content on Twitter related to hikikomori in the Japanese language and to assess Twitter users’ engagement with that content.

**Methods:**

We conducted a mixed methods analysis of a random sample of 4940 Japanese tweets from February to August 2018 using a hashtag (#hikikomori). Qualitative content analysis included examination of the text of each tweet, development of a codebook, and categorization of tweets into relevant codes. For quantitative analysis (n=4859 tweets), we used bivariate and multivariate logistic regression models, adjusted for multiple comparisons, and estimated the predicted probabilities of tweets receiving engagement (likes or retweets).

**Results:**

Our content analysis identified 9 codes relevant to tweets about hikikomori: *personal anecdotes*, *social support*, *marketing*, *advice*, *stigma*, *educational opportunities*, *refuge (ibasho)*, *employment opportunities*, and *medicine and science*. Tweets about *personal anecdotes* were the most common (present in 2747/4859, 56.53% of the tweets), followed by *social support* (902/4859, 18.56%) and *marketing* (624/4859, 12.84%). In the adjusted models, tweets coded as *stigma* had a lower predicted probability of likes (−33 percentage points, 95% CI −42 to −23 percentage points; *P*<.001) and retweets (−11 percentage points, 95% CI −18 to −4 percentage points; *P*<.001), *personal anecdotes* had a lower predicted probability of retweets (−8 percentage points, 95% CI −14 to −3 percentage points; *P*=.002), *marketing* had a lower predicted probability of likes (−13 percentage points, 95% CI −21 to −6 percentage points; *P*<.001), and *social support* had a higher predicted probability of retweets (+15 percentage points, 95% CI 6-24 percentage points; *P*=.001), compared with all tweets without each of these codes.

**Conclusions:**

Japanese tweets about hikikomori reflect a unique array of topics, many of which have not been identified in prior research and vary in their likelihood of receiving engagement. Tweets often contain personal stories of hikikomori, suggesting the potential to identify individuals with hikikomori through Twitter.

## Introduction

### Background

Hikikomori is a form of severe social withdrawal, initially described in Japan in the 1990s, and since the 2010s, it has been increasingly reported in other countries around the globe, including the Western world [1,2]. Individuals with hikikomori are described as people who shut themselves in their homes for months and even years, with minimal interaction with society and little to no participation in school or the workforce [3]. Hikikomori can cause significant distress to the affected individuals and is often associated with psychiatric disorders [4,5]. It has also been considered a major socioeconomic and public health concern in Japan for years, with an estimated prevalence of approximately 1% [6,7].

A longstanding area of debate is whether hikikomori constitutes (or is a manifestation of) psychopathology versus sociological phenomena such as nonmainstream lifestyle preferences, cultural marginalization [8], or nonconforming reactions to societal constraints [9]. To an extent, hikikomori represents psychopathology, and additional issues include how to diagnose and treat it [7].

The nature of hikikomori makes affected individuals a hard-to-reach population [10] in terms of research and intervention efforts. Although hikikomori was described in Japan much before the *digital revolution* of the 2000s, the internet, social media, and web-based gaming have radically changed the way people interact [11]. This may be particularly relevant among individuals with hikikomori, a *hidden population* that might be spending a considerable amount of time on the internet for entertainment and social interaction [12]. Indeed, the relationships among internet use, video gaming, social media use, and hikikomori have been studied in Japan [13]. Given this, the *online world* has been proposed as an accessible gateway to reach and support individuals with hikikomori [10,14].

Social media platforms, including Twitter [15], Facebook [16], and Instagram [17] have been increasingly harnessed for health research. Twitter, a popular microblogging platform mainly based in short text posts (*tweets*), counts on >300 million users worldwide [18] and provides open access to its public contents. Health research on Twitter has included exploration of content in the public conversation regarding health conditions and treatments, engagement of users (reach of general public, recruitment of research subjects, and intervention on target populations), and real time epidemiological surveillance [15] (these applications of internet and social media-based data have been named *Infodemiology* and *infoveillance*) [19].

Twitter can be especially useful for health research in Japan, as it is the most popular social media platform in this country [20], with 51.9 million users as of October 2020 (in absolute number of Twitter users worldwide, Japan is only behind the United States, which has 68.7 million users) [18]. We previously reported findings based on analysis of tweets containing the hashtag #hikikomori [10]; the study found that tweets depicted hikikomori as either *not a problem* (eg, as a lifestyle or a nonconcerning behavior) or as a medical or social problem. Tweets with scientific content and tweets mentioning hikikomori in countries other than Japan showed significantly higher user engagement than those without these topics. However, the study was limited in sample size and only included tweets in 5 Western languages (English, Italian, Spanish, Catalan, and French).

### Objectives

The objective of this study is to analyze Japanese language tweets related to hikikomori. Our two primary research questions are as follows: (1) What are the main types of content among Japanese language tweets related to hikikomori? (2) What tweets result in the most engagement (as measured by users’ retweets and likes)?

## Methods

### Study Design and Overview

In this mixed methods study [21], we used concurrent collection and analysis of qualitative and quantitative social media data to better understand hikikomori. Qualitative data and analysis focused on content analysis of publicly available tweets about hikikomori in the Japanese language, whereas quantitative data and analysis focused on metrics of engagement with the tweets contained in the content analysis.

Translation (Japanese to English) was done by bilingual research team members (ART and MPJT), with backtranslation (English to Japanese) by native Japanese speakers (TH and RK). This study was approved by the University of Navarra Research Ethics Committee (ID: 2018.36-mod1) and the Veterans Affairs Portland Health System Research and Development Committee (ID: 4524). We used publicly available tweets, which are subject to universal access according to Twitter’s terms of service [22].

### Data Collection and Curation

**Figure 1:**
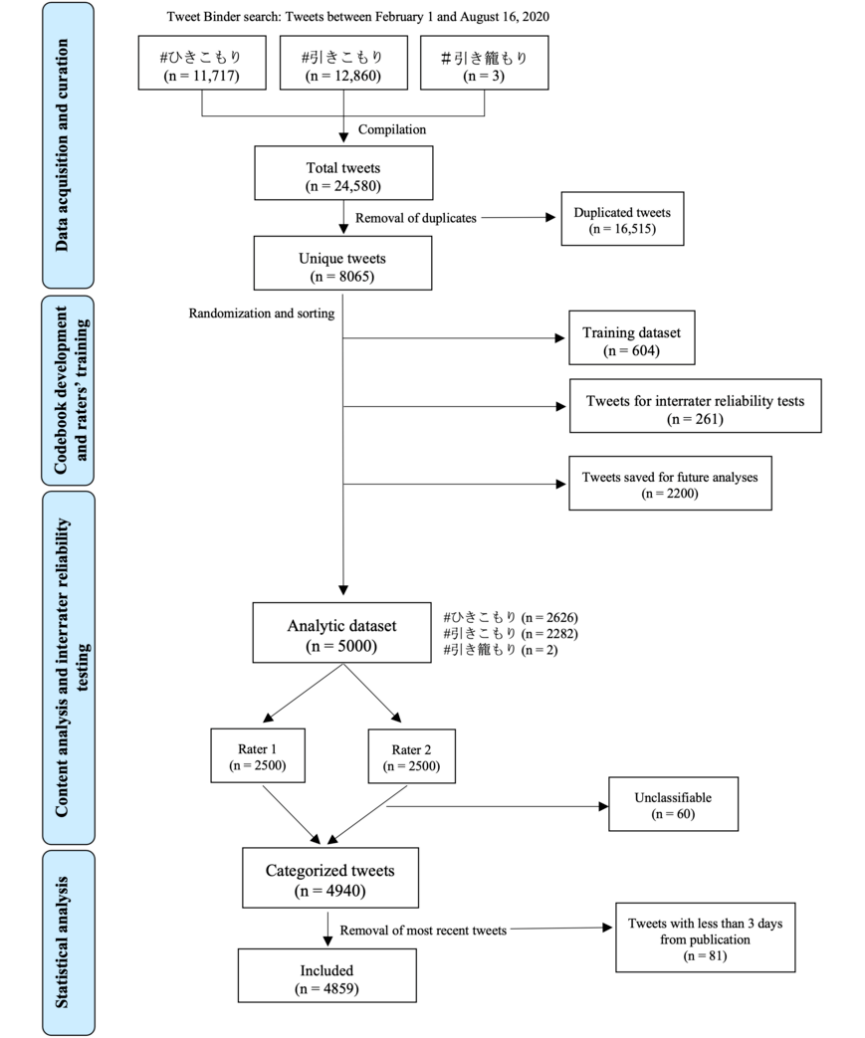
Flowchart summarizing the steps in data collection and analysis.

Figure 1 presents a flowchart summarizing the steps in data collection and analysis, along with the number of tweets included and excluded in each step. We used the Tweet Binder engine for the identification and collection of tweets. As described in our previous studies on Twitter content analysis, [10,23-25], Tweet Binder uses the Twitter provider Firehose via the tool Gnip (Gnip Inc) to access 100% of the public tweets matching a specific query, whereas some other search engines based on Twitter’s application programing interface can only access small samples [15,26].

We included tweets that met the following criteria: (1) were public (ie, not posted as *protected* by users); (2) contained any of the 3 hashtags, each representing a way to transcribe the term *hikikomori* in Japanese (#ひきこもりor #引きこもり or #引き籠り); (3) were posted between February 1 and August 16, 2018; and (4) contained any text in Japanese besides the hashtag itself. The exclusion criteria were as follows: (1) the majority of text in the tweet was in another language besides Japanese or (2) tweets only contained a link or picture without any text. Extracted information also included metadata (date of tweets, contributors’ usernames and number of followers, number of likes and retweets, frequently associated hashtags, etc). We merged the full data for each of the tweets containing 1 of the 3 hashtags into 1 data set, removed duplicates, and randomized the order of tweets in the data set.

### Content Analysis: Codebook Development and Training

Training was provided by research team members (VPS and ART) experienced in content analysis and codebook development [10,16,24]. There were 2 primary coders (TH and RK), with a third research team member (MPJT) assisting with adjudication of coding disagreements.

We first created a *training data set* of 604 tweets for content exploration and for training coders. Both coders looked at the text of each tweet independently, being blind to its metadata (username and date), to identify both hypothesis-driven codes (ie, types of contents previously identified in our study on hikikomori in Western languages [10]: *hikikomori not as a problem*, *medicine and science*, *personal anecdotes*, *social*, *scientific reference*, and *hikikomori out of Japan*) and data-driven codes (new types of contents). Tweets could be coded into multiple codes when appropriate, although the assignment of a single code was preferred.

A codebook was developed using an iterative process through regular team meetings; the final codebook comprised 9 codes that fit all the main topics present in the data set and for which the interrater reliability (IRR), as measured by agreement percentages, was very high (>95%). Table 1, which lists these codes and provides definitions and examples, was used as a reference for coders in the following step.

**Table 1:** Codebook for content analysis (N=5000 tweets).^a^

Code	Definition	Examples of tweets
Unclassifiable	Tweets with insufficient information to be coded. These typically included brief tweets or tweets with seemingly random content with little relevance to hikikomori.	“I’m in the intensive camp trying to get a driving license, I can’t stand it anymore #event #smartphone #driverslicense #camp #Iwannagohome #hikikomori #ubearable (URL).” [Tweet ID 1695]
Personal anecdotes	Tweets describing experiences with hikikomori. These can either be from people who self-identify as hikikomori (first person stories) or comments about others thought to have hikikomori (second or third person stories).	“A quiet morning. He^b^ hasn’t come into the living room. Perhaps he’s having a restful sleep? 1 year and several months ago Even if he had medications he couldn’t sleep. It was difficult for him to fall asleep alone. Now, even without medication, he falls asleep just like this. I hope he sleeps a lot...#depression #hikikomori #schoolrefusal.” [Tweet ID 4796]
Social support	Tweets about resources that may provide social support, such as online or face-to-face support groups or hotlines for people affected by hikikomori.	“Self-help group for those who are unemployed or on leave (URL) # Self-helpgroup “Intersection”#Unemployed #On leave #Returntowork #Depression #Hikikomori #Socialparticipation #Self-helpgroup #Createanibasho #Urawa^c^#counseling #NEET^d^ #Psychiatry.” [Tweet ID 1156] “Good evening, this is Akebonobashi^c^ Independence Training Center. Are you troubled by #Hikikomori #NEET #developmental disorder #domestic violence? Do discuss it with us! (URL).” [Tweet ID 1320]
Marketing	Tweets advertising or offering services to individuals with hikikomori (note: if the service being marketed was a job offer or schooling or educational opportunity, they were coded using those codes instead).	“There aren’t many people with a PC who aren’t doing this you know? It’s what happens when you try too hard...lol^e^ ⇒ (URL) #Sidejob #Millionaire #NEET #Hikikomori.” [Tweet ID 1011]
Advice	Tweet offering suggestions, recommendations, or advice for individuals with hikikomori.	“Today, discussed “listening communication” at the “trouble with returning to work café.” While communication tends to emphasize “speaking,” actually “listening” can also be important! Perhaps this approach can be effective for people who have problems with communication? #support with return to work #youthsupport #ibasho #Hikikomori (URL).” [Tweet ID 172]
Stigma	Tweets using hikikomori as a pejorative word or insult.	“Colorful, small-sized clothes are worn by both older women, and younger ladies in their 20s. Even if both of these groups have a slim and short physique, I think there are clothes that are suitable for a certain age. It’s like washing everyone’s clothes together on the weekend.^f^ #Cigarettesmell #Noise #Annoyingbehavior #Condominiumresident #Okagami^c^ #Hikikomori #Makingyoung #disgusting #Aunt.” [Tweet ID 1021]
Educational opportunities	Tweets about schooling options or other educational opportunities for individuals with a diagnosis of hikikomori.	“Kyoto or Osaka correspondence school. A school in Kyoto, which is eligible for a secure study Support System. (URL) #School refusal #Hikikomori.” [Tweet ID 677]
Refuge (*Ibasho*)	Tweet that describes or offers a refuge, respite, or other safe space for people (including those affected by hikikomori). In Japanese, the term *ibasho* is used.	“Hi there! Today at my ibasho, I had a day of art 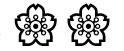 A bit like winter? Using felt to make a mini cushion! Feel like it turned out pretty good 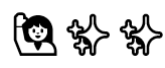 #Tokyo #tokyo #tokyo^g^ #sugamo^c^ #sugamo^c,g^ #hikikomori...(URL).” [Tweet ID 399]
Employment opportunities	Tweets offering jobs for individuals with hikikomori.	“Ocomail^h^ is in production! #Starfish* #myjobistofind people #recruitment #Occupation #Kagawa^c^ #Kochi^c^ #Tokushima^c^ #Ehime^c^ #Shikoku^c^ #LGBT #Hikikomori #Elderlypeople #Foreigners #Mother #Disabledpeople #Careerchange #Jobhunting #Job #Staffrecruitment #Recruitment #Mid-career recruitment #Ocomail” [Tweet ID 155]
Medicine and science	Tweet related to the epidemiology, psychopathology, diagnosis, research, or treatment of hikikomori. Tweets with an explicit reference to a scientific publication, government document, or other official source are also included here.	“20xx news from cabinet office: Hikikomori (40-64 yrs old) A national survey...‘rarely leaves their own room,’ ‘travels only to nearby convenience stores’ → counselling with Hikikomori (URL) #cabinet office #Hikikomori #nationalsurvey (URL).” [Tweet ID 11,384] “The average age of hikikomori is 34.4 years, with an average duration of 11.8 years. The ages appear to be increasing. ⇒ The average age of actual hikikomori is 34.4 years old, with an average duration of hikikomori is 11 years 8 months. Both of which were the highest ever in an official survey. #Hikikomori (URL).” [Tweet ID 3212]

^a^This table presents the definitions and examples of the codes in our codebook. Spacing between lines and paragraphs, if present in the original tweets, was removed to shorten the length of the table. Hyperlinks, when present in the original tweet, were removed in these examples (we leave [URL] to indicate that a hyperlink was present in the original tweet). All hashtags (starting with #) were translated into English unless they used unique concepts without appropriate English equivalents.

^b^The tweet does not specify what gender the person in question is; the male pronoun is used only for the purposes of this translation.

^c^Toponym.

^d^NEET: not in employment, education, or training.

^e^The letter “W” in the original text is thought to represent an onomatopoeia for the sound of laughter. On the internet, it is used similar to its English equivalent of *lol* (ie, *laugh out loud*).

^f^It is standard practice in Japan to do one’s laundry separate from others, particularly as people commonly live in shared accommodation.

^g^The Japanese language comprises of multiple writing systems; here, the same toponyms are spelled in different hashtags using different writing systems.

^h^*Ocomail* is a popular Japanese company that specializes in shipping locally grown Japanese rice overseas.

### Content Analysis: Categorization of Tweets

An independent subsample of 5000 tweets (*analytic data set*) was used for content analysis, and the newly developed codebook was applied. Each coder independently examined the texts of 50% (2500/5000) tweets. For each tweet, raters were instructed to determine whether it fit the inclusion criteria (60/5000, 1.2% tweets were *unclassifiable* and excluded) and code it for the presence or absence of each of the 9 codes.

To ensure acceptable IRR and prevent *coder drift* [27], both raters also coded 3 batches of tweets from an independent subset (*IRR data set*), 1 batch per week during the first 3 weeks of the content analyses. The lead investigator (VPS) monitored the IRR for these batches and provided interim feedback when appropriate.

### Statistical Analyses: IRR and Engagement Metrics

Statistical analyses were conducted using Stata 16 (StataCorp) and included calculations of IRR, descriptive figures of the distribution of tweets by codes, and analysis of engagement metrics.

We calculated the agreement percentages to assess the IRR for the 261 double-coded tweets from the *IRR data set*, which were used for coder training and not used during content analyses. Agreement percentages (presented as last batch of double coding/average across batches of double coding) were 79.21%/77.84% for *personal anecdotes*, 91.09%/90.68% for *social support*, 87.13%/85.71% for *marketing*, 91.09%/90.68% for *advice*, 92.08%/89.44% for *stigma*, 98.02%/96.89% for *educational opportunities*, 98.02%/96.89% for *refuge* (*ibasho*), 99.01%/98.76% for *employment opportunities*, and 99.01%/99.38% for *medicine and science*. We use agreement percentages over κ coefficients, as the latter underestimates agreement when the *prevalence* values (in our case, number of tweets) for a category or code are too low [27].

As for users’ engagement metrics, we analyzed likes and retweets for each code in the analytic data set. Previous research has estimated that most users’ engagement with social media content occurs within a week after posting [28]. Therefore, we further restricted our analytic sample to tweets that had at least 3 days of follow-up between posting and data collection, thereby excluding 1.64% (81/4940) tweets.

Bivariate logistic regression models were used to test for the association between tweets and the presence of a code and receiving at least one like or retweet. Multivariate models tested the same association with adjustment for (1) user’s number of tweets in our analytic sample, (2) user’s number of followers, and (3) number of days between posting and data collection. All models were clustered by the user. Results were presented as proportion points difference (also understood as difference in predicted probabilities between tweets with and without the code) rather than model coefficients for ease of interpretation [29]. Critical values for Bonferroni correction for multiple comparisons were calculated by dividing the α level (.05) by the number of hypotheses (9) and applied to all results (critical value *P*<.005). These regression models were preferred over linear regressions for numbers of likes and retweets because of the uneven distribution of these numbers among tweets.

## Results

As detailed in the flowchart presented in Figure 1, of the 8065 tweets collected by the Tweet Binder tool, 4940 (61.25%) and 4859 (60.25%) unique tweets were included in the qualitative content and quantitative data analysis, respectively. A total of 1680 unique users contributed to those tweets, with an average of 1 tweet per user (median 1; IQR 1-2); only 54 users (3.21%) contributed >10 tweets.

### Content of Tweets That Reference Hikikomori

The codebook applied to the analytic data set included 9 codes, 1 of which was hypothesis-driven from our previous research [10] and the remaining 8 were data-driven based on the exploration of tweets in the *training data set*:

1. Hypothesis-driven code: *Medicine and science* included tweets related to epidemiological, therapeutic, or research aspects of hikikomori understood as a pathology (eg, a tweet about a published research paper on hikikomori).
2. Data-driven codes: *Marketing*, *employment opportunities*, and *educational opportunities* included different kinds of offers apparently targeted at people with hikikomori. *Social support* and *refuge* (*ibasho*) comprised tweets promoting resources to help people with hikikomori, such as online or onsite *social support* groups, hotlines, or *ibasho*—a Japanese concept referring to designated spaces of psychological comfort for people in distress [30]. *Personal anecdotes* were related to stories of people describing hikikomori symptoms or behaviors with or without negative connotations (*stigma*) or helpful information (*advice*).

As mentioned earlier, definitions and examples are available in Table 1.

**Figure 2:**
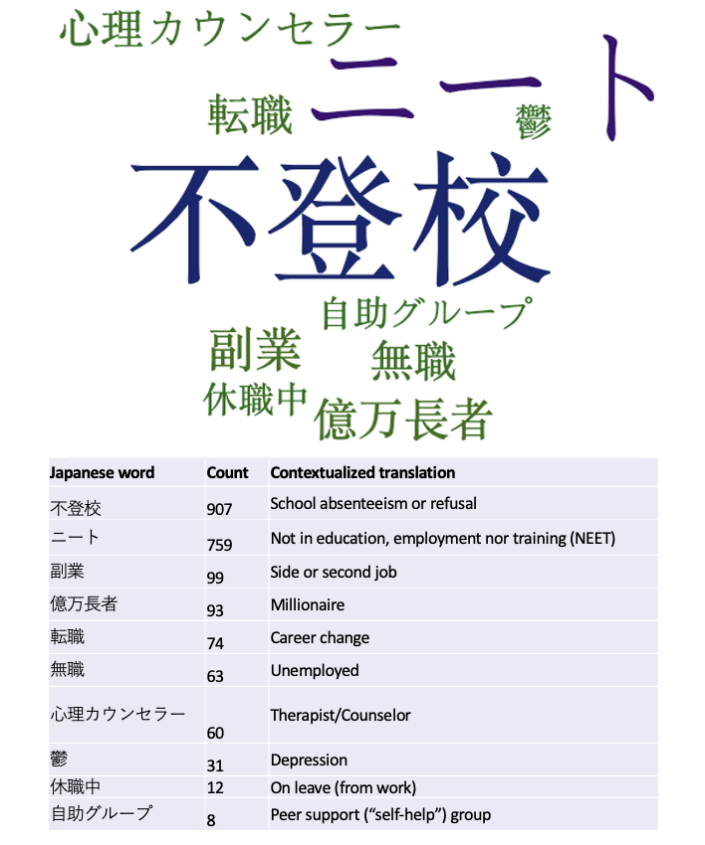
Word cloud illustrating the 10 most frequently used hashtags in the tweets analyzed.

The 10 most frequently used hashtags are represented in Figure 2. The most common hashtags were related to education and employment (#school absenteeism or #refusal and #NEET, an acronym used to refer to people *not in education, employment, nor training*), whereas some less frequent ones were related to mental health and support.

### Distribution of Tweets by Codes

The tweet contents were unevenly distributed across the codes. Of the 4859 tweets, the code *personal anecdotes* was present in 2747 (56.53%), whereas *social support* was present in 902 (18.56%) tweets, and *marketing* was present in 624 (12.84%) tweets. To note, *medicine and science* was the code with the least tweets (31/4859, 0.63%). Complete figures on the number and percentages of tweets per code are presented in Table 2, along with the descriptive figures for likes and retweets.

**Table 2:** Descriptive characteristics of the tweets included in the analysis by code (N=4859).^a^

Code	Tweets, n (%)	Likes			Retweets	
		At least one^b^, n (%)	Median^c^ (IQR)	At least one^b^, n (%)		Median^c^ (IQR)
Personal anecdotes	2747 (56.53)	1318 (48)	3 (1-7)	436 (15.9)		1 (1-3)
Social support	902 (18.56)	276 (30.6)	2 (1-4)	211 (23.4)		1 (1-3)
Marketing	624 (12.84)	199 (31.9)	2 (1-5)	106 (17)		1 (1-2)
Advice	281 (5.78)	93 (33.1)	2 (1-5)	60 (21.4)		1 (1-3)
Stigma	166 (3.42)	12 (7.2)	1 (1-3)	9 (5.4)		1 (1-1)
Educational opportunities	129 (2.65)	37 (28.7)	2 (1-5)	21 (16.3)		2 (1-3)
Refuge (*Ibasho*)	86 (1.77)	40 (46.5)	4 (1-7)	30 (34.9)		2 (1-4)
Employment opportunities	82 (1.69)	28 (34.2)	3 (1-13)	21 (25.6)		3 (1-15)
Medicine and science	31 (0.64)	16 (51.6)	2 (1-3)	7 (22.6)		4 (2-7)

^a^For each code, the total number of tweets and retweets (n) and relative proportions (%) are provided. The total number of tweets in the first column may add to more than the total number of tweets that we have analyzed because 1 tweet could be coded into multiple codes.

^b^Among tweets in the code, n (%) with at least one like (or retweet).

^c^Among tweets in the code which had at least one like (or retweet), median (IQR) of the number of likes (or retweets).

### Engagement Metrics by Codes

Approximately half of all tweets in *medicine and science*, *personal anecdotes*, and *refuge* (*ibasho*) codes received at least one like (16/31, 51.6%; 1318/2747, 48%; and 40/86, 46.5%, respectively), whereas one-third (30/86, 35%) of tweets in *refuge* (*ibasho*)’ and approximately one-quarter of tweets in *employment opportunities* (21/82, 26%) and *social support* (211/902, 23.4%) received at least one retweet. Tweets with the code *stigma* had the lowest probability of having at least one like (12/166, 7.2%) or 1 retweet (9/166, 5.4%; Table 2).

Results from logistic regression analyses with clustering by user are presented in Table 3 (for likes) and Table 4 (for retweets). In unadjusted models, tweets with the *stigma* code had a significantly lower predicted probability of receiving likes (−35 percentage points, 95% CI −45 to −25 percentage points; *P*<.001) and receiving retweets (−13 percentage points, 95% CI −20 to −7 percentage points; *P*<.001) compared with all tweets without that code. Tweets coded as *personal anecdotes* had a significantly higher predicted probability of receiving likes (+16 percentage points, 95% CI 3-29 percentage points; *P*=.02) compared with all tweets without that code. No other associations between codes and being liked or retweeted were significant in the unadjusted models.

**Table 3:** Association between content analysis codes and receiving at least one like (N=4859 tweets) using logistic regression with adjustment for covariates and clustering by user.^a^

Code	Estimated probability (95% CI) by tweet content				*P* value
	Tweets without code (%)	Tweets with code (%)	Difference (percentage points)		
Personal anecdotes	35.4 (30.3 to 40.5)	44.9 (36.5 to 53.3)	9.5 (0.5 to 18.5)	.04^b^	
Social support	41 (34.1 to 47.9)	41.2 (32.9 to 49.4)	0.2 (−12.6 to 12.9)	.98	
Marketing	42.9 (37.6 to 48.1)	29.5 (19.8 to 39.3)	−13.3 (−20.8 to −5.9)	**<**.001^b,c^	
Advice	41.1 (35.2 to 47.1)	39.7 (26.3 to 53.1)	−1.4 (−16.9 to 14.0)	.86	
Stigma	42.1 (36.4 to 47.7)	9.5 (2.6 to 16.3)	−32.6 (−41.9 to −23.3)	<.001^b,c^	
Educational opportunities	41.5 (35.8 to 47.1)	27.5 (16.7 to 38.4)	−13.9 (−25.4 to −2.4)	.02^b,c^	
Refuge (*Ibasho*)	41.1 (35.5 to 46.6)	40.5 (16.4 to 64.7)	−0.5 (−24.5 to 23.5)	.97	
Employment opportunities	41.2 (35.6 to 46.8)	33.7 (21.9 to 45.4)	−7.5 (−20.2 to 5.1)	.24	
Medicine and science	41 (35.5 to 46.6)	42.4 (23.7 to 61)	1.3 (−17.5 to 20.2)	.89	

^a^Results are expressed as the difference in predicted probability of at least one like between tweets with and without the code, wherein a positive value indicates a higher probability of receiving a like among tweets with the code present compared with tweets without the code. Models adjusted for (1) number of user tweets in the data set, (2) number of followers for the user, and (3) number of days between posting and the data collection date.

^b^Significance at critical value *P*<.05.

^c^Significant at the Bonferroni-adjusted critical value *P*<.006.

**Table 4:** Association between content analysis code and receiving at least one retweet (N=4859 tweets) using logistic regression with adjustment for covariates and clustering by user.^a^

Code	Estimated probability (95% CI) by tweet content				*P* value
	Tweets without code (%)	Tweets with code (%)	Difference (percentage points)		
Personal anecdotes	23.2 (19.1 to 27.3)	14.9 (11.2 to 18.6)	−8.3 (−13.6 to −3.1)	.002^b,c^	
Social support	16.1 (12.9 to 19.3)	31.4 (23.6 to 39.3)	15.3 (6.3 to 24.3)	.001^b,c^	
Marketing	18.7 (15.6 to 21.8)	15.2 (10 to 20.3)	−3.5 (−8.2 to 1.2)	.14	
Advice	17.8 (14.6 to 21)	25.6 (18.7 to 32.5)	7.8 (−0.3 to 16.0)	.06	
Stigma	18.5 (15.4 to 21.6)	7.5 (1.7 to 13.4)	−11.0 (−17.6 to −4.3)	.001^b,c^	
Educational opportunities	18.3 (15.2 to 21.3)	15.7 (6.9 to 24.5)	−2.5 (−11.5 to 6.4)	.58	
Refuge (*Ibasho*)	18 (15 to 20.9)	29 (8.8 to 4.9)	11 (−8.7 to 30.7)	.27	
Employment opportunities	18.1 (15 to 21.1)	24.8 (11.1 to 38.5)	6.8 (−7.1 to 20.6)	.34	
Medicine and science	18.2 (15.1 to 21.2)	17.1 (3.8 to 30.4)	−1.1 (−14.4 to 12.2)	.87	

^a^Results are expressed as the difference in predicted probability of at least one retweet between tweets with and without the code, where a positive value indicates a higher probability of receiving a retweet among tweets with the code present compared with tweets without the code. Models adjusted for (1) number of user tweets in the data set, (2) number of followers for the user, and (3) number of days between posting and data collection date.

^b^Significance at critical value *P*<.05.

^c^Significant at the Bonferroni-adjusted critical value *P*<.006.

In adjusted models, the associations between *stigma* and lower predicted probability of being liked (−33 percentage points, 95% CI −42 to −23 percentage points; *P*<.001) and retweeted (−11 percentage points, 95% CI −18 to −4 percentage points; *P*<.001) remained highly significant, whereas the association of *personal anecdotes* with a higher predicted probability of being liked lost significance. In contrast, several associations that were not significant in the unadjusted models became significant after adjustment. Tweets in *marketing* had a significantly lower predicted probability of receiving likes (−13 percentage points, 95% CI −21 to −6 percentage points; *P*<.001), tweets with *personal anecdotes* had a significantly lower predicted probability of receiving retweets (−8 percentage points, 95% CI −14.0 to −3 percentage points; *P*=.002), and tweets with *social support* had a significantly higher probability of receiving retweets (+15 percentage points, 95% CI 6-24 percentage points; *P*=.001), compared with all tweets without each of these codes. These associations were statistically significant at the false discovery rate critical value (*P*<.017) to account for multiple comparisons. Other associations that were significant at the level of *P*<.05 are presented in Tables 3 and 4.

## Discussion

### Principal Findings

Our mixed method analysis of nearly 5000 Japanese language tweets revealed a unique array of topics discussed in relation to hikikomori, many of which have not been identified in prior studies. Personal anecdotes about hikikomori predominated, suggesting that individual Twitter users are willing to share their personal stories and experiences with hikikomori on social media. School absenteeism (*futoko*) and withdrawal from the education system and labor force (*not in employment, education, or training*) were also commonly associated with the hikikomori hashtag, adding corroboration from Twitter data that these 2 concepts are closely linked to the lives of people with hikikomori and frequently discussed in Japan [8,31]. Engagement (retweets and likes) varied by tweet content, but tweets with stigmatizing content received consistently lower engagement.

To the best of our knowledge, this report presents the first application of social media research to a data set of Japanese tweets related to hikikomori. This study builds upon our previous study of tweets with #hikikomori in several Western languages [10], both by examining a significantly larger data set and also by identifying a distinct set of topics within the Japanese Twitter discourse on hikikomori. In contrast to our prior study, this study revealed that tweets in Japanese tend to relate to personal stories (*personal anecdotes*) of hikikomori, as well as *marketing* (in many cases, presented as *click-bait* [32]) and social support opportunities targeting individuals with hikikomori. These findings support the suspicion that social media may indeed be a refuge for individuals with hikikomori and serve as a place where they can find social support [10,14].

It is noteworthy that the code *medicine and science* was by far the least identified in the Japanese data set, accounting for <1% of the tweets, in contrast to our previous study on tweets in Western languages, where these contents were present in 42.22% of classifiable tweets [10]. This suggests the existence of cross-cultural differences in the way hikikomori is conceived and discussed by the general public in Japan versus Western countries. Although hikikomori seems to be a term more integrated in popular culture and a part of one’s identity in Japan, Western countries tend to view it as a worrisome behavior and related to mental health issues.

Our discovery of stigmatizing tweets eliciting *negative* public engagement (lower predicted probability of retweets and likes) is worth discussing. Stigma, a social phenomenon involving negative attitudes toward people with certain characteristics or conditions, markedly affects people with mental disorders [33]. Given the potential role that social media plays in the perpetuation of misinformation, stereotypes, and hateful speech, psychiatric research in this area has particularly focused on stigma [34]; examples include studies on psychosis and schizophrenia [24,35,36], bipolar disorder [37], and the depiction of mental disorders by mass media [38]. The infrequency of stigmatizing Twitter content related to hikikomori and the relative lack of engagement with such content are hopeful findings.

A final point for discussion of our results relates to our research question about the differential patterns of public engagement (retweets and likes) generated by each of the topics. Engagement with social media content, apart from being a marker of visibility, may reflect the public’s interest, perceptions, and behavior [23,28,39]. One possible explanation for the pattern of a higher probability of likes but a lower predicted probability for retweets observed for *personal anecdotes* tweets is that Twitter users may show solidarity with the person disclosing hikikomori but less willingness to publicly share and endorse those personal stories with their own followers.

Our social media research on hikikomori, the results of which are presented in our previous study of Western language tweets [10] and this study, constitutes the first application of Twitter content analyses to this phenomenon. To contextualize our study in the scientific literature, 2 aspects are worth noting. On the one hand, our methods were built on previous social media studies in the area of health [24,25,28,37] and incorporated innovations based on our own hypotheses and the retrieved data. Social media research is relatively young, and the preferred methodology is subject to change. In contrast, recent hikikomori research has paid more attention to the interplay among social withdrawal, smartphones and technology, internet use and addiction, and social media, where causal relationships seem difficult to untangle [11,13]. Further work is needed to fine-tune and replicate Twitter analysis methods in health research, as well as to study social media use by people with hikikomori, examining the patterns of use, contents they consume and generate and their influence on them and on the general public, and avenues for research and public health interventions to reach and support them.

### Limitations

The main limitations of this study are as follows: (1) hyperlinks that were included in the original tweets were not analyzed, which limited the ability to understand the full context of the tweets; (2) other tweets potentially related to hikikomori may have been missed if they used other hashtags not captured in our data set; (3) although high overall, the IRR was variable across codes and weaker in some of them, especially in *personal anecdotes* code; (4) metrics of engagement (likes and retweets) may have been influenced by unknown or unmeasurable confounding factors (eg, characteristics of the user posting the content, factors related to the user’s followers, and other contextual factors).

### Conclusions

In conclusion, Japanese tweets that are related to hikikomori are abundant and contain a wide array of topics. Engagement patterns varied but stigmatizing and marketing content were generally less likely to receive engagement, whereas personal stories and social support showed some evidence of being more likely to receive engagement. Future research to better understand the characteristics that make some tweets more likely to elicit reactions [40,41], their significance [42], and the intriguing ways in which retweets and likes converge and diverge [24] would be helpful. Our findings can inform Twitter content to potentially identify and connect with this hard-to-reach population.

## Abbreviations

IRR: interrater reliability
